# Supplementary material for: mRNA-1273 bivalent (original and Omicron) COVID-19 vaccine effectiveness against COVID-19 outcomes in the United States
Source: Nat Commun. 2023 Sep 20;14:5851. doi: 10.1038/s41467-023-41537-7 (PMC10511551; doi:10.1038/s41467-023-41537-7)
Supplement: Supplementary file 1 — Supplementary Information [file 41467_2023_41537_MOESM1_ESM.pdf]

mRNA-1273 bivalent (Original and Omicron) COVID-19 vaccine effectiveness against COVID-19 outcomes in the United States

Authors: Hung Fu Tseng,[1,2] Bradley K. Ackerson,[1] Lina S. Sy,[1] Julia E. Tubert,[1] Yi Luo,[1] Sijia Qiu,[1] Gina S. Lee,[1] Katia J. Bruxvoort,[1,3] Jennifer H. Ku,[1] Ana Florea,[1] Harpreet S. Takhar,[1] Radha Bathala,[1] Cindy Ke Zhou,[4] Daina B. Esposito,[4] Morgan A. Marks,[4] Evan J. Anderson,[4] Carla A. Talarico,[4,5] Lei Qian[1]

Affiliations:

[1]Department of Research and Evaluation, Kaiser Permanente Southern California, Pasadena, CA 91101, USA

[2]Department of Health Systems Science, Kaiser Permanente Bernard J. Tyson School of Medicine, Pasadena, CA 91101, USA

[3]Department of Epidemiology, University of Alabama at Birmingham, Birmingham, AL 35233, USA

[4]Moderna Inc., Cambridge, MA 02139, USA

[5]AstraZeneca, 1 Medimmune Way, Gaithersburg, MD 20878, USA

Table of Contents

Supplementary Table 1. Comparison of baseline characteristics between individuals in the bivalent (original and Omicron BA.4/BA.5) mRNA-1273 COVID-19 vaccine cohort and the COVID-19 unvaccinated cohort. 3

Supplementary Table 2. Incidence rate, hazard ratio, and relative effectiveness of the bivalent (original and Omicron BA.4/BA.5) mRNA-1273 COVID-19 vaccine in preventing hospitalization for COVID-19, overall and by subgroups (≥2 monovalent mRNA vaccine group as comparator). 9

Supplementary Table 3. Incidence rate, hazard ratio, and effectiveness of the bivalent (original and Omicron BA.4/BA.5) mRNA-1273 COVID-19 vaccine in preventing hospitalization for COVID-19, overall and by subgroups (COVID-19 unvaccinated group as comparator). 12

Supplementary Table 4. Incidence rate, hazard ratio, and relative effectiveness of the bivalent (original and Omicron BA.4/BA.5) mRNA-1273 COVID-19 vaccine in preventing medically attended SARS-CoV-2 infection and COVID-19 hospital death (≥2 monovalent mRNA vaccine group as comparator). 15

Supplementary Table 5. Incidence rate, hazard ratio, and effectiveness of the bivalent (original and Omicron BA.4/BA.5) mRNA-1273 COVID-19 vaccine in preventing medically attended SARS-CoV-2 infection and COVID-19 hospital death (COVID-19 unvaccinated group as comparator). 16

Supplementary Table 6. Strengthening the Reporting of Observational Studies in Epidemiology (STROBE) checklist. 17

Supplementary Table 1. Comparison of baseline characteristics between individuals in the bivalent (original and Omicron BA.4/BA.5) mRNA-1273 COVID-19 vaccine cohort and the COVID-19 unvaccinated cohort.

	Bivalent Vaccine Group	COVID-19 Unvaccinated Group[a]	Total	p value	Absolute Standardized Difference
	**N=290292**	**N=204655**	**N=494947**		
Age at index date, years				<0.01	0.34
mean (sd)	58.67 (17.53)	52.55 (18.04)	56.14 (18.00)		
median	62	53	58		
Q1, Q3	46, 72	40, 66	44, 70		
min, max	6, 10	6, 11	6, 11		
Age at index date, years, n (%)				<0.01	0.32
6-17	2715 (0.9%)	2715 (1.3%)	5430 (1.1%)		
18-44	63953 (22.0%)	57434 (28.1%)	121387 (24.5%)		
45-64	96293 (33.2%)	85376 (41.7%)	181669 (36.7%)		
65-74	73258 (25.2%)	37473 (18.3%)	110731 (22.4%)		
≥75	54073 (18.6%)	21657 (10.6%)	75730 (15.3%)		
Sex, n (%)				<0.01	0.02
Female	157727 (54.3%)	112732 (55.1%)	270459 (54.6%)		
Male	132565 (45.7%)	91923 (44.9%)	224488 (45.4%)		
Race/Ethnicity, n (%)				<0.01	0.27
Non-Hispanic White	114740 (39.5%)	85828 (41.9%)	200568 (40.5%)		
Non-Hispanic Black	23517 (8.1%)	16662 (8.1%)	40179 (8.1%)		
Hispanic	82547 (28.4%)	67279 (32.9%)	149826 (30.3%)		
Non-Hispanic Asian	50129 (17.3%)	17459 (8.5%)	67588 (13.7%)		
Other/Unknown	19359 (6.7%)	17427 (8.5%)	36786 (7.4%)		

	Bivalent Vaccine Group	COVID-19 Unvaccinated Group[a]	Total	p value	Absolute Standardized Difference
	N=290292	N=204655	N=494947		
Body mass index[b], kg/m^2, n (%)				<0.01	0.24
<18.5	5233 (1.8%)	4291 (2.1%)	9524 (1.9%)		
18.5 - <25	75876 (26.1%)	44238 (21.6%)	120114 (24.3%)		
25 - <30	89285 (30.8%)	56946 (27.8%)	146231 (29.5%)		
30 - <35	53028 (18.3%)	37658 (18.4%)	90686 (18.3%)		
35 - <40	24218 (8.3%)	17156 (8.4%)	41374 (8.4%)		
40 - <45	10083 (3.5%)	6790 (3.3%)	16873 (3.4%)		
≥45	6432 (2.2%)	4104 (2.0%)	10536 (2.1%)		
Unknown	26137 (9.0%)	33472 (16.4%)	59609 (12.0%)		
Smoking[b], n (%)				<0.01	0.19
No	216879 (74.7%)	142550 (69.7%)	359429 (72.6%)		
Yes	55588 (19.1%)	38415 (18.8%)	94003 (19.0%)		
Unknown	17825 (6.1%)	23690 (11.6%)	41515 (8.4%)		
Charlson comorbidity score[c], n (%)				<0.01	0.33
0	162456 (56.0%)	145154 (70.9%)	307610 (62.2%)		
1	50534 (17.4%)	27666 (13.5%)	78200 (15.8%)		
≥2	77302 (26.6%)	31835 (15.6%)	109137 (22.1%)		
Frailty index[c]				<0.01	0.16
mean (sd)	0.12 (0.03)	0.12 (0.03)	0.12 (0.03)		
median	0.11	0.11	0.11		
Q1, Q3	0.10, 0.14	0.10, 0.13	0.10, 0.13		
min, max	0.04, 0.41	0.04, 0.41	0.04, 0.41		

	Bivalent Vaccine Group	COVID-19 Unvaccinated Group[a]	Total	p value	Absolute Standardized Difference
	N=290292	N=204655	N=494947		
Frailty index[c], n (%)				<0.01	0.35
Quartile 1	77937 (26.8%)	45784 (22.4%)	123721 (25.0%)		
Quartile 2	55217 (19.0%)	68551 (33.5%)	123768 (25.0%)		
Quartile 3	74608 (25.7%)	49109 (24.0%)	123717 (25.0%)		
Quartile 4, most frail	82530 (28.4%)	41211 (20.1%)	123741 (25.0%)		
Chronic diseases[c], n (%)					
Kidney disease	28145 (9.7%)	10538 (5.1%)	38683 (7.8%)	<0.01	0.17
Heart disease	14069 (4.8%)	7477 (3.7%)	21546 (4.4%)	<0.01	0.06
Lung disease	34966 (12.0%)	18063 (8.8%)	53029 (10.7%)	<0.01	0.11
Liver disease	11715 (4.0%)	5996 (2.9%)	17711 (3.6%)	<0.01	0.06
Diabetes	58781 (20.2%)	24753 (12.1%)	83534 (16.9%)	<0.01	0.22
Immunocompromised status, n (%)				<0.01	0.11
Yes	12338 (4.3%)	4788 (2.3%)	17126 (3.5%)		
HIV/AIDS	1894	295	2189		
Leukemia, lymphoma, congenital and other immunodeficiencies, asplenia/hyposplenia	5252	2299	7551		
Organ transplant	1205	325	1530		
Immunosuppressant medications	6343	2511	8854		
Autoimmune conditions[c], n (%)				<0.01	0.08

	Bivalent Vaccine Group	COVID-19 Unvaccinated Group[a]	Total	p value	Absolute Standardized Difference
	**N=290292**	**N=204655**	**N=494947**		
Yes	12183 (4.2%)	5650 (2.8%)	17833 (3.6%)		
Rheumatoid arthritis	5206	2549	7755		
Inflammatory bowel disease	2063	971	3034		
Psoriasis and psoriatic arthritis	4641	1930	6571		
Multiple sclerosis	614	357	971		
Systemic lupus erythematosus	807	435	1242		
Pregnant at index date, n (%)				<0.01	0.06
Yes	1200 (0.4%)	1759 (0.9%)	2959 (0.6%)		
1st trimester	280	503	783		
2nd trimester	462	610	1072		
3rd trimester	458	646	1104		
History of SARS-CoV-2 infection[d], n (%)				<0.01	0.19
Yes	69405 (23.9%)	66117 (32.3%)	135522 (27.4%)		
≤180 days	31592	13526	45118		
181-365 days	20310	25857	46167		
>365 days	17503	26734	44237		
History of SARS-CoV-2 molecular test[d], n (%)	194122 (66.9%)	118191 (57.8%)	312313 (63.1%)	<0.01	0.19
Number of outpatient and virtual visits[c], n (%)				<0.01	0.56
0	12737 (4.4%)	28594 (14.0%)	41331 (8.4%)		
1-4	68261 (23.5%)	78391 (38.3%)	146652 (29.6%)		

	Bivalent Vaccine Group	COVID-19 Unvaccinated Group[a]	Total	p value	Absolute Standardized Difference
	N=290292	N=204655	N=494947		
5-10	87609 (30.2%)	50454 (24.7%)	138063 (27.9%)		
≥11	121685 (41.9%)	47216 (23.1%)	168901 (34.1%)		
Number of Emergency Department visits[c], n (%)				<0.01	0.08
0	244234 (84.1%)	166007 (81.1%)	410241 (82.9%)		
1	32951 (11.4%)	26830 (13.1%)	59781 (12.1%)		
≥2	13107 (4.5%)	11818 (5.8%)	24925 (5.0%)		
Number of hospitalizations[c], n (%)				<0.01	0.05
0	276000 (95.1%)	192324 (94.0%)	468324 (94.6%)		
1	11132 (3.8%)	9575 (4.7%)	20707 (4.2%)		
≥2	3160 (1.1%)	2756 (1.3%)	5916 (1.2%)		
Preventive care[c], n (%)	251979 (86.8%)	111295 (54.4%)	363274 (73.4%)	<0.01	0.76
Medicaid, n (%)	17571 (6.1%)	23186 (11.3%)	40757 (8.2%)	<0.01	0.19
Neighborhood median household income, n (%)				<0.01	0.17
< $40,000	8206 (2.8%)	8353 (4.1%)	16559 (3.3%)		
$40,000-$59,999	44119 (15.2%)	36800 (18.0%)	80919 (16.3%)		
$60,000-$79,999	62206 (21.4%)	50238 (24.5%)	112444 (22.7%)		
≥$80,000	175589 (60.5%)	108468 (53.0%)	284057 (57.4%)		
Unknown	172 (0.1%)	796 (0.4%)	968 (0.2%)		
Concomitant vaccination[e], n (%)	50729 (17.5%)	N/A	N/A	N/A	N/A
Antiviral therapy[f], n (%)				<0.01	0.09

	Bivalent Vaccine Group	COVID-19 Unvaccinated Group[a]	Total	p value	Absolute Standardized Difference
	N=290292	N=204655	N=494947		
Yes	4094 (1.4%)	1126 (0.6%)	5220 (1.1%)		
Nirmatrelvir/ritonavir	4058	1113	5171		
Molnupiravir	36	10	46		
Remdesivir	3	3	6		
Medical center area[g], n (%)				<0.01	0.52
Month of index date, n (%)				<0.01	0.03
September 2022	45806 (15.8%)	32853 (16.1%)	78659 (15.9%)		
October 2022	88322 (30.4%)	59849 (29.2%)	148171 (29.9%)		
November 2022	87272 (30.1%)	62030 (30.3%)	149302 (30.2%)		
December 2022	68892 (23.7%)	49923 (24.4%)	118815 (24.0%)		

χ^2 tests were used for categorical variables and two-sided, two-sample t tests were used for continuous variables.
Min, minimum; max, maximum; N/A, not applicable; Q, quartile; sd, standard deviation
[a]Not all bivalent-vaccinated individuals were matched to an COVID-19 unvaccinated comparator. Unmatched bivalent-vaccinated individuals were kept in the analysis.
[b]Defined in the two years prior to index date.
[c]Defined in the one year prior to index date.
[d]Defined based on all available medical records from March 1, 2020 to index date.
[e]Among subjects with concomitant vaccines received with the bivalent mRNA-1273 vaccine: influenza vaccine (89.6%), shingles vaccine (9.1%), pneumococcal vaccine (2.9%), Tdap (2.6%), and other vaccine (1.3%).
[f]Defined during follow-up.
[g]Frequency and percent for the 19 medical center areas not shown.

Supplementary Table 2. Incidence rate, hazard ratio, and relative effectiveness of the bivalent (original and Omicron BA.4/BA.5) mRNA-1273 COVID-19 vaccine in preventing hospitalization for COVID-19, overall and by subgroups (≥2 monovalent mRNA vaccine group as comparator).

	Bivalent vaccine group				≥2 monovalent mRNA vaccine group				Hazard Ratio (95% CI)		rVE (95% CI)	
**Hospitalization for COVID-19**	**N**	**Number of cases**	**Number of person years**	**Incidence per 1000 person-years (95% CI)**	**N**	**Number of cases**	**Number of person years**	**Incidence per 1000 person-years (95% CI)**	**Unadjusted**	**Adjusted[a]**	**Unadjusted**	**Adjusted[a]**
Overall	290292	160	59235.05	2.70 (2.31-3.15)	580584	646	76386.05	8.46 (7.83-9.14)	0.34 (0.28-0.40)	0.30 (0.25-0.36)	66.3 (59.9-71.7)	70.3 (64.0-75.4)
Age at index date, years[b]												
6-17	2715	0	418	N/A	5430	0	698.51	N/A	N/A	N/A	N/A	N/A
18-44	63953	2	13001.62	0.15 (0.04-0.62)	127906	10	20439.67	0.49 (0.26-0.91)	0.31 (0.07-1.44)	0.22 (0.04-1.15)	68.6 (-30.5-93.1)	78.4 (-12.7-95.9)
45-64	96293	19	19008.44	1.00 (0.64-1.57)	192586	49	26447.6	1.85 (1.40-2.45)	0.56 (0.33-0.95)	0.44 (0.25-0.77)	44.5 (5.4-67.4)	56.2 (22.5-75.2)
65-74	73258	41	15402.42	2.66 (1.96-3.62)	146516	148	16799.18	8.81 (7.50-10.35)	0.32 (0.23-0.46)	0.31 (0.21-0.45)	67.7 (54.1-77.2)	69.3 (55.2-79.0)
≥75	54073	98	11404.56	8.59 (7.05-10.47)	108146	439	12001.09	36.58 (33.31-40.17)	0.24 (0.19-0.30)	0.29 (0.22-0.36)	75.7 (69.7-80.6)	71.4 (63.6-77.6)
Sex												
Female	157727	81	32124.22	2.52 (2.03-3.13)	315454	303	41338.32	7.33 (6.55-8.20)	0.36 (0.28-0.46)	0.34 (0.26-0.44)	64.0 (53.9-71.9)	66.5 (56.0-74.5)
Male	132565	79	27110.83	2.91 (2.34-3.63)	265130	343	35047.72	9.79 (8.80-10.88)	0.32 (0.25-0.41)	0.27 (0.20-0.35)	68.3 (59.5-75.2)	73.4 (65.3-79.7)
Race/Ethnicity												
Non-Hispanic White	114740	77	24473.05	3.15 (2.52-3.93)	229480	309	29162.19	10.60 (9.48-11.85)	0.32 (0.25-0.41)	0.30 (0.23-0.40)	68.2 (59.1-75.3)	70.0 (60.2-77.4)
Non-Hispanic Black	23517	17	4520.19	3.76 (2.34-6.05)	47034	71	5720.44	12.41 (9.84-15.66)	0.31 (0.18-0.53)	0.26 (0.15-0.47)	68.8 (46.7-81.7)	73.6 (53.0-85.1)
Hispanic	82547	44	15843.06	2.78	165094	166	23173.71	7.16	0.40	0.36	59.8	63.8

	Bivalent vaccine group				≥2 monovalent mRNA vaccine group				Hazard Ratio (95% CI)		rVE (95% CI)	
Hospitalization for COVID-19	N	Number of cases	Number of person years	Incidence per 1000 person-years (95% CI)	N	Number of cases	Number of person years	Incidence per 1000 person-years (95% CI)	Unadjusted	Adjusted[a]	Unadjusted	Adjusted[a]
				(2.07-3.73)				(6.15-8.34)	(0.29-0.56)	(0.25-0.52)	(43.9-71.2)	(48.4-74.6)
Non-Hispanic Asian	50129	21	10450.58	2.01 (1.31-3.08)	100258	84	12661.84	6.63 (5.36-8.22)	0.31 (0.19-0.51)	0.24 (0.14-0.40)	68.6 (49.0-80.6)	76.3 (59.9-86.0)
Immunocompromised status												
Yes[c]	12338	30	2553.68	11.75 (8.21-16.80)	19991	83	2289.59	36.25 (29.23-44.95)	0.33 (0.22-0.51)	0.35 (0.22-0.56)	66.7 (49.3-78.2)	64.7 (44.0-77.7)
No	277954	130	56681.38	2.29 (1.93-2.72)	560593	563	74096.45	7.60 (7.00-8.25)	0.32 (0.26-0.39)	0.29 (0.23-0.35)	68.1 (61.3-73.6)	71.3 (64.5-76.7)
History of SARS-CoV-2 infection												
Yes[d]	69405	21	13177.97	1.59 (1.04-2.44)	155360	82	21153.77	3.88 (3.12-4.81)	0.42 (0.26-0.68)	0.39 (0.23-0.66)	58.1 (32.1-74.1)	60.7 (33.8-76.7)
No	220887	139	46057.08	3.02 (2.56-3.56)	425224	564	55232.27	10.21 (9.40-11.09)	0.31 (0.26-0.38)	0.29 (0.24-0.35)	68.5 (62.1-73.9)	71.1 (64.6-76.5)
Months of follow-up												
0-<1 month	290292	56	23761.66	2.36 (1.81-3.06)	580584	388	39288.55	9.88 (8.94-10.91)	0.24 (0.18-0.31)	0.25 (0.19-0.34)	76.2 (68.5-82.0)	74.9 (66.4-81.3)
1-<2 months	265962	45	18967.95	2.37 (1.77-3.18)	378308	197	24055.89	8.19 (7.12-9.42)	0.29 (0.21-0.40)	0.24 (0.17-0.35)	70.9 (59.7-78.9)	75.8 (65.3-83.2)
2-<3 months	190879	46	11685.44	3.94 (2.95-5.26)	211800	52	10801.58	4.81 (3.67-6.32)	0.84 (0.56-1.24)	0.41 (0.25-0.67)	16.5 (-19.6-43.9)	58.9 (33.5-74.6)
≥3 months[e]	92435	13	4413.99	2.95 (1.71-5.07)	62428	9	2138.48	4.21 (2.19-8.09)	0.82 (0.35-1.92)	0.20 (0.07-0.57)	18.2 (-48.0-65.2)	79.6 (43.2-92.7)
Number of monovalent vaccines prior to index date[f]												
2 doses	11493	3	2203.2	1.36	135437	149	22354.78	6.67	0.22	0.23	78.0	77.3

Hospitalization for COVID-19	Bivalent vaccine group				≥2 monovalent mRNA vaccine group				Hazard Ratio (95% CI)		rVE (95% CI)	
	N	Number of cases	Number of person years	Incidence per 1000 person-years (95% CI)	N	Number of cases	Number of person years	Incidence per 1000 person-years (95% CI)	Unadjusted	Adjusted[a]	Unadjusted	Adjusted[a]
				(0.44-4.22)				(5.68-7.83)	(0.07-0.69)	(0.07-0.72)	(31.0-93.0)	(27.6-92.9)
3 doses	144052	48	28340.77	1.69 (1.28-2.25)	287077	311	39692.37	7.84 (7.01-8.76)	0.23 (0.17-0.31)	0.30 (0.22-0.40)	77.2 (69.0-83.2)	70.4 (59.6-78.3)
≥4 doses	134747	109	28691.09	3.80 (3.15-4.58)	158070	186	14338.9	12.97 (11.24-14.98)	0.29 (0.22-0.37)	0.28 (0.22-0.36)	71.3 (63.3-77.5)	71.7 (63.7-78.0)
Time between latest monovalent vaccine and index date[g]												
≤180 days	83060	83	19923.17	4.17 (3.36-5.17)	134392	142	13192.87	10.76 (9.13-12.69)	0.40 (0.30-0.52)	0.36 (0.27-0.48)	60.3 (47.6-70.0)	64.0 (51.7-73.2)
181-365 days	165276	64	33439.32	1.91 (1.50-2.45)	274298	281	37735.09	7.45 (6.62-8.37)	0.27 (0.20-0.35)	0.22 (0.17-0.29)	73.3 (65.0-79.7)	78.0 (71.1-83.3)
>365 days	41956	13	5872.56	2.21 (1.29-3.81)	171894	223	25458.09	8.76 (7.68-9.99)	0.25 (0.14-0.43)	0.26 (0.15-0.46)	75.5 (57.1-86.0)	73.6 (53.7-85.0)

CI, confidence interval; rVE, relative vaccine effectiveness
[a]Adjusted for covariates age group, sex, race/ethnicity, index date (in months), history of SARS-CoV-2 infection, number of outpatient and virtual visits, preventive care, number of monovalent vaccines prior to index date, time between latest monovalent vaccine and index date, and antiviral therapy. Medical center area removed from adjustment set due to lack of model convergence.
[b]Adjusted for continuous age (in years) in addition to covariates above.
[c]Adjusted for immunocompromising sub-conditions in addition to covariates above.
[d]Adjusted for time since prior SARS-CoV-2 infection in addition to covariates above.
[e]Maximum follow-up time was 4.57 months.
[f]Time between latest monovalent vaccine and index date removed from adjustment set due to lack of model convergence.
[g]Number of monovalent vaccines prior to index date removed from adjustment set due to lack of model convergence.
When the hazard ratio or its 95% CI was >1, the rVE or its 95% CI was transformed as ([1/hazard ratio] – 1) × 100

Supplementary Table 3. Incidence rate, hazard ratio, and effectiveness of the bivalent (original and Omicron BA.4/BA.5) mRNA-1273 COVID-19 vaccine in preventing hospitalization for COVID-19, overall and by subgroups (COVID-19 unvaccinated group as comparator).

	Bivalent vaccine group				COVID-19 unvaccinated group				Hazard Ratio (95% CI)		VE (95% CI)	
**Hospitalization for COVID-19**	**N**	**Number of cases**	**Number of person years**	**Incidence per 1000 person-years (95% CI)**	**N**	**Number of cases**	**Number of person years**	**Incidence per 1000 person-years (95% CI)**	**Unadjusted**	**Adjusted[a]**	**Unadjusted**	**Adjusted[a]**
Overall	290292	160	59235.05	2.70 (2.31-3.15)	204655	341	39937.04	8.54 (7.68-9.49)	0.32 (0.26-0.38)	0.17 (0.14-0.21)	68.4 (61.9-73.8)	82.8 (78.8-86.0)
Age at index date, years[b]												
6-17	2715	0	418	N/A	2715	0	411.76	N/A	N/A	N/A	N/A	N/A
18-44[c,d]	63953	2	13001.62	0.15 (0.04-0.62)	57434	5	11004.34	0.45 (0.19-1.09)	0.34 (0.07-1.74)	0.13 (0.02-0.89)	66.2 (-42.7-93.4)	87.5 (10.5-98.2)
45-64	96293	19	19008.44	1.00 (0.64-1.57)	85376	43	16201.03	2.65 (1.97-3.58)	0.38 (0.22-0.65)	0.22 (0.12-0.39)	62.2 (35.1-77.9)	78.4 (61.2-87.9)
65-74	73258	41	15402.42	2.66 (1.96-3.62)	37473	91	7774.11	11.71 (9.53-14.38)	0.23 (0.16-0.33)	0.18 (0.12-0.27)	77.3 (67.2-84.3)	82.1 (73.2-88.0)
≥75	54073	98	11404.56	8.59 (7.05-10.47)	21657	202	4545.79	44.44 (38.71-51.01)	0.19 (0.15-0.25)	0.16 (0.12-0.21)	80.7 (75.4-84.8)	84.3 (79.4-88.1)
Sex												
Female	157727	81	32124.22	2.52 (2.03-3.13)	112732	192	22033.95	8.71 (7.56-10.04)	0.29 (0.22-0.37)	0.18 (0.14-0.24)	71. (62.6-77.7)	81.8 (75.8-86.3)
Male	132565	79	27110.83	2.91 (2.34-3.63)	91923	149	17903.09	8.32 (7.09-9.77)	0.35 (0.27-0.46)	0.16 (0.12-0.22)	65.0 (54.1-73.4)	83.7 (77.7-88.0)
Race/Ethnicity												
Non-Hispanic White	114740	77	24473.05	3.15 (2.52-3.93)	85828	183	17737.23	10.32 (8.93-11.93)	0.30 (0.23-0.40)	0.16 (0.12-0.21)	69.5 (60.3-76.7)	84.0 (78.5-88.1)
Non-Hispanic Black	23517	17	4520.19	3.76 (2.34-6.05)	16662	44	3043.7	14.46 (10.76-19.43)	0.26 (0.15-0.45)	0.14 (0.08-0.26)	74.0 (54.5-85.2)	86.1 (74.4-92.5)
Hispanic	82547	44	15843.06	2.78	67279	82	12510.61	6.55	0.42	0.23	57.6	77.0

	Bivalent vaccine group				COVID-19 unvaccinated group				Hazard Ratio (95% CI)		VE (95% CI)	
**Hospitalization for COVID-19**	**N**	**Number of cases**	**Number of person years**	**Incidence per 1000 person-years (95% CI)**	**N**	**Number of cases**	**Number of person years**	**Incidence per 1000 person-years (95% CI)**	**Unadjusted**	**Adjusted[a]**	**Unadjusted**	**Adjusted[a]**
				(2.07-3.73)				(5.28-8.14)	(0.29-0.61)	(0.15-0.34)	(38.9-70.6)	(65.5-84.6)
Non-Hispanic Asian	50129	21	10450.58	2.01 (1.31-3.08)	17459	27	3260	8.28 (5.68-12.08)	0.24 (0.14-0.42)	0.11 (0.06-0.21)	76.1 (57.7-86.5)	89.2 (78.9-94.5)
Immunocompromised status												
Yes[c,e]	12338	30	2553.68	11.75 (8.21-16.80)	4788	25	952.23	26.25 (17.74-38.85)	0.45 (0.26-0.76)	0.28 (0.16-0.51)	55.3 (24.0-73.7)	71.8 (48.8-84.5)
No	277954	130	56681.38	2.29 (1.93-2.72)	199867	316	38984.81	8.11 (7.26-9.05)	0.28 (0.23-0.35)	0.16 (0.13-0.20)	71. (65.4-77.0)	84.1 (80.1-87.4)
History of SARS-CoV-2 infection												
Yes[f]	69405	21	13177.97	1.59 (1.04-2.44)	66117	58	12742.28	4.55 (3.52-5.89)	0.35 (0.21-0.58)	0.32 (0.18-0.55)	64. (42.1-78.7)	68.3 (45.4-81.6)
No	220887	139	46057.08	3.02 (2.56-3.56)	138538	283	27194.75	10.41 (9.26-11.69)	0.29 (0.24-0.35)	0.16 (0.13-0.20)	71.0 (64.5-76.4)	84.3 (80.3-87.5)
Months of follow-up												
0-<1 month	290292	56	23761.66	2.36 (1.81-3.06)	204655	124	16474.69	7.53 (6.31-8.98)	0.31 (0.23-0.43)	0.15 (0.11-0.22)	68.7 (57.1-77.2)	84.8 (78.5-89.3)
1-<2 months	265962	45	18967.95	2.37 (1.77-3.18)	181530	115	12788.96	8.99 (7.49-10.80)	0.26 (0.19-0.37)	0.14 (0.10-0.21)	73.6 (62.7-81.3)	85.6 (79.0-90.2%)
2-<3 months	190879	46	11685.44	3.94 (2.95-5.26)	126732	84	7627.96	11.01 (8.89-13.64)	0.36 (0.25-0.51)	0.23 (0.15-0.35)	64.2 (48.7-75.0)	76.8 (65.0-84.6)
≥3 months[g]	92435	13	4413.99	2.95 (1.71-5.07)	58823	18	2797.68	6.43 (4.05-10.21)	0.46 (0.23-0.94)	0.25 (0.11-0.56)	54.1 (6.2-77.5)	75.5 (43.8-89.3)

CI, confidence interval; VE, vaccine effectiveness
[a]Adjusted for covariates age group, sex, race/ethnicity, index date (in months), body mass index, smoking, Charlson comorbidity score, frailty index, kidney disease, lung disease, diabetes, immunocompromised status, history of SARS-CoV-2 infection, history of SARS-CoV-2 molecular

test, number of outpatient and virtual visits, preventive care, Medicaid, and antiviral therapy. Neighborhood median household income and medical center area removed from adjustment set due to lack of model convergence.
[b]Adjusted for continuous age (in years) in addition to covariates above.
[c]Smoking removed from adjustment set due to lack of model convergence.
[d]Kidney disease, lung disease, and immunocompromised status removed from adjustment set due to lack of model convergence.
[e]Adjusted for immunocompromising sub-conditions in addition to covariates above.
[f]Adjusted for time since prior SARS-CoV-2 infection in addition to covariates above.
[g]Maximum follow-up time was 4.57 months.
When the hazard ratio or its 95% CI was >1, the VE or its 95% CI was transformed as ([1/hazard ratio] – 1) × 100.

Supplementary Table 4. Incidence rate, hazard ratio, and relative effectiveness of the bivalent (original and Omicron BA.4/BA.5) mRNA-1273 COVID-19 vaccine in preventing medically attended SARS-CoV-2 infection and COVID-19 hospital death (≥2 monovalent mRNA vaccine group as comparator).

	Bivalent vaccine group				≥2 monovalent mRNA vaccine group				Hazard Ratio (95% CI)		rVE (95% CI)	
**Outcomes**	**N**	**Number of cases**	**Number of person years**	**Incidence per 1000 person-years (95% CI)**	**N**	**Number of cases**	**Number of person years**	**Incidence per 1000 person-years (95% CI)**	**Unadjusted**	**Adjusted[a]**	**Unadjusted**	**Adjusted[a]**
Medically attended SARS-CoV-2 infection												
All care settings	290292	3221	58867.97	54.72 (52.86-56.64)	580584	4964	75886.22	65.41 (63.62-67.26)	0.84 (0.80-0.88)	0.64 (0.61-0.67)	16.0 (12.1-19.7)	35.9 (32.7-39.0)
Emergency department and urgent care	290292	855	59149.21	14.45 (13.52-15.46)	580584	2083	76214.31	27.33 (26.18-28.53)	0.55 (0.51-0.60)	0.45 (0.41-0.49)	45.0 (40.4-49.2)	55.0 (50.8-58.8)
COVID-19 hospital death[b]	290292	10	59252.1	0.17 (0.09-0.31)	580584	59	76449.67	0.77 (0.60-1.00)	0.22 (0.11-0.44)	0.17 (0.08-0.36)	77.7 (56.4-88.6)	82.7 (63.7-91.7)

CI, confidence interval; rVE, relative vaccine effectiveness

[a]Adjusted for covariates age group, sex, race/ethnicity, index date (in months), history of SARS-CoV-2 infection, number of outpatient and virtual visits, preventive care, number of monovalent vaccines prior to index date, time between latest monovalent vaccine and index date, and medical center area.

[b]Adjusted for antiviral therapy in addition to covariates above. Medical center area removed from adjustment set due to lack of model convergence.

Supplementary Table 5. Incidence rate, hazard ratio, and effectiveness of the bivalent (original and Omicron BA.4/BA.5) mRNA-1273 COVID-19 vaccine in preventing medically attended SARS-CoV-2 infection and COVID-19 hospital death (COVID-19 unvaccinated group as comparator).

	Bivalent vaccine group				COVID-19 unvaccinated group				Hazard Ratio (95% CI)		VE (95% CI)	
**Outcomes**	**N**	**Number of cases**	**Number of person years**	**Incidence per 1000 person-years (95% CI)**	**N**	**Number of cases**	**Number of person years**	**Incidence per 1000 person-years (95% CI)**	**Unadjusted**	**Adjusted[a]**	**Unadjusted**	**Adjusted[a]**
Medically attended SARS-CoV-2 infection												
All care settings	290292	3221	58867.97	54.72 (52.86-56.64)	204655	1578	39779.99	39.67 (37.76-41.67)	1.38 (1.30-1.46)	0.89 (0.83-0.96)	-27.3 (-31.6--22.8)	10.7 (4.4-16.6)
Emergency department and urgent care	290292	855	59149.21	14.45 (13.52-15.46)	204655	815	39872.1	20.44 (19.08-21.89)	0.71 (0.64-0.78)	0.45 (0.40-0.50)	29.3 (22.1-35.7)	55.4 (50.3-60.1)
COVID-19 hospital death[b]	290292	10	59252.1	0.17 (0.09-0.31)	204655	35	39972.07	0.88 (0.63-1.22)	0.19 (0.09-0.39)	0.10 (0.05-0.22)	80.9 (61.5-90.5)	89.7 (77.7-95.2)

CI, confidence interval; VE, vaccine effectiveness

[a]Adjusted for covariates age group, sex, race/ethnicity, index date (in months), body mass index, smoking, Charlson comorbidity score, frailty index, kidney disease, lung disease, diabetes, immunocompromised status, history of SARS-CoV-2 infection, history of SARS-CoV-2 molecular test, number of outpatient and virtual visits, preventive care, Medicaid, neighborhood median household income, and medical center area.

[b]Adjusted for antiviral therapy in addition to covariates above. Neighborhood median household income and medical center area removed from adjustment set due to lack of model convergence.

When the hazard ratio or its 95% CI was >1, the VE or its 95% CI was transformed as ([1/hazard ratio] – 1) × 100.

Supplementary Table 6. Strengthening the Reporting of Observational Studies in Epidemiology (STROBE) Statement
STROBE Statement—checklist of items that should be included in reports of observational studies.

Pages and manuscript line numbers refer to the final submitted version.

Section	Item No.	Recommendation	Implementation
**Title and abstract**	1	(*a*) Indicate the study's design with a commonly used term in the title or the abstract	Abstract
		(*b*) Provide in the abstract an informative and balanced summary of what was done and what was found	Abstract
**Introduction**			
Background/rationale	2	Explain the scientific background and rationale for the investigation being reported	Introduction
Objectives	3	State specific objectives, including any prespecified hypotheses	Introduction
**Methods**			
Study design	4	Present key elements of study design early in the paper	Abstract, Introduction, and Methods
Setting	5	Describe the setting, locations, and relevant dates, including periods of recruitment, exposure, follow-up, and data collection	Methods
Participants	6	(*a*) *Cohort study*—Give the eligibility criteria, and the sources and methods of selection of participants. Describe methods of follow-up<br>*Case-control study*—Give the eligibility criteria, and the sources and methods of case ascertainment and control selection. Give the rationale for the choice of cases and controls<br>*Cross-sectional study*—Give the eligibility criteria, and the sources and methods of selection of participants	Methods
		(*b*) *Cohort study*—For matched studies, give matching criteria and number of exposed and unexposed<br>*Case-control study*—For matched studies, give matching criteria and the number of controls per case	Methods

Section	Item No.	Recommendation	Implementation
Variables	7	Clearly define all outcomes, exposures, predictors, potential confounders, and effect modifiers. Give diagnostic criteria, if applicable	Methods
Data sources/ measurement	8*	For each variable of interest, give sources of data and details of methods of assessment (measurement). Describe comparability of assessment methods if there is more than one group	Methods
Bias	9	Describe any efforts to address potential sources of bias	Methods
Study size	10	Explain how the study size was arrived at	Methods and Figure 1
Quantitative variables	11	Explain how quantitative variables were handled in the analyses. If applicable, describe which groupings were chosen and why	Methods
Statistical methods	12	(*a*) Describe all statistical methods, including those used to control for confounding	Methods
		(*b*) Describe any methods used to examine subgroups and interactions	Methods
		(*c*) Explain how missing data were addressed	Methods
		(*d*) *Cohort study*—If applicable, explain how loss to follow-up was addressed<br>*Case-control study*—If applicable, explain how matching of cases and controls was addressed<br>*Cross-sectional study*—If applicable, describe analytical methods taking account of sampling strategy	Methods
		(*e*) Describe any sensitivity analyses	n/a
**Results**			
Participants	13*	(a) Report numbers of individuals at each stage of study—eg numbers potentially eligible, examined for eligibility, confirmed eligible, included in the study, completing follow-up, and analysed	Results and Figure 1
		(b) Give reasons for non-participation at each stage	Result and Figure 1
		(c) Consider use of a flow diagram	Results and Figure 1

Section	Item No.	Recommendation	Implementation
Descriptive data	14*	(a) Give characteristics of study participants (eg demographic, clinical, social) and information on exposures and potential confounders	Results, Figure 1, Table 1, and Table S1
		(b) Indicate number of participants with missing data for each variable of interest	Results, Table 1, and Table S1
		(c) *Cohort study*—Summarise follow-up time (eg, average and total amount)	Results, Table S2, Table S3, and Figure 3
Outcome data	15*	*Cohort study*—Report numbers of outcome events or summary measures over time	Results, Figure 2, Figure 4, and Table S2-S5
		Case-control study—Report numbers in each exposure category, or summary measures of exposure	n/a
		Cross-sectional study—Report numbers of outcome events or summary measures	n/a
Main results	16	(*a*) Give unadjusted estimates and, if applicable, confounder-adjusted estimates and their precision (eg, 95% confidence interval). Make clear which confounders were adjusted for and why they were included	Results, Figure 2, Figure 4, and Table S2-S5
		(*b*) Report category boundaries when continuous variables were categorized	Results, Table 1, and Table S1
		(*c*) If relevant, consider translating estimates of relative risk into absolute risk for a meaningful time period	n/a
Other analyses	17	Report other analyses done—eg analyses of subgroups and interactions, and sensitivity analyses	Results, Figure 3, and Table S2-S3
**Discussion**			
Key results	18	Summarise key results with reference to study objectives	Discussion
Limitations	19	Discuss limitations of the study, taking into account sources of potential bias or imprecision. Discuss both direction and magnitude of any potential bias	Discussion
Interpretation	20	Give a cautious overall interpretation of results considering objectives, limitations, multiplicity of analyses, results from similar studies, and other relevant evidence	Discussion

Section	Item No.	Recommendation	Implementation
Generalisability	21	Discuss the generalisability (external validity) of the study results	Discussion
**Other information**			
Funding	22	Give the source of funding and the role of the funders for the present study and, if applicable, for the original study on which the present article is based	Funding statement

*Give information separately for cases and controls in case-control studies and, if applicable, for exposed and unexposed groups in cohort and cross-sectional studies.

Note: An Explanation and Elaboration article discusses each checklist item and gives methodological background and published examples of transparent reporting. The STROBE checklist is best used in conjunction with this article (freely available on the Web sites of PLoS Medicine at http://www.plosmedicine.org/, Annals of Internal Medicine at http://www.annals.org/, and Epidemiology at http://www.epidem.com/). Information on the STROBE Initiative is available at www.strobe-statement.org.
